# Supplementary material for: In Silico Screening of Bacteriocin Gene Clusters within a Set of Marine Bacillota Genomes
Source: Int J Mol Sci. 2024 Feb 22;25(5):2566. doi: 10.3390/ijms25052566 (PMC10931772; doi:10.3390/ijms25052566)
Supplement: Supplementary file 1 [file ijms-25-02566-s001.zip › ijms-2838454-supplementary.pdf]

Supplementary Materials for:

In Silico Screening of Bacteriocin Gene Clusters within a Set of Marine *Bacillota* Genomes

Rabeb Teber [1] and Shuichi Asakawa [1,2,*]

[1] Laboratory of Aquatic Molecular Biology and Biotechnology, Department of Aquatic Bioscience, Graduate School of Agricultural and Life Sciences, The University of Tokyo, Bunkyo, Tokyo 113-8657, Japan; rabeb.teber@gmail.com

[2] Signal Peptidome Research Laboratory, Department of Aquatic Bioscience, Graduate School of Agricultural and Life Sciences, The University of Tokyo, Bunkyo, Tokyo 113-8657, Japan

* Correspondence: asakawa@mail.ecc.u-tokyo.ac.jp

Table S1. Detailed information about the 51 chosen *Bacillota* strains

**Strain**	**Isolation source**	**Isolation country**	**RefSeq assembly accession number**	**NCBI Refseq accession number or GenBank accession number**
Oceanobacillus iheyensis HTE831	Deep sea mud	Japan	GCF_000011245.1	NC_004193.1
Geobacillus kaustophilus HTA426	Deep sea sediment	Pacific Ocean	GCF_000009785.1	NC_006510.1, NC_006509.1
Bacillus sp. Pc3	Sea water	Antarctica	GCF_000815145.1	NZ_CP010406.1
Geobacillus sp. 12AMOR1	Marine sediment	Norway	Unknown	CP011832.1: CP011833.1
Bacillus infantis NRRL B-14911	Sea water	USA	GCF_000473245.1	NC_022524.1
Bacillus weihaiensis Alg07	Brown seaweed	China	GCF_001889165.1	NZ_CP016020.1: NZ_CP016021.1
Bacillus safensis KCTC 12796BP	Marine sponge	South Korea	GCF_001895885.1	NZ_CP018197.1: NZ_CP018198.1

Bacillus velezensis 9912D	Sediment	China	GCF_001857985.1	NZ_CP017775.1: NZ_CP017776.1
Bacillus cereus CC-1	Marine sediment	China	GCF_002290105.1	NZ_CP023179.1: NZ_CP023180.1: NZ_CP023181.1: NZ_CP023182.1: NZ_CP023183.1
Halobacillus mangrovi KTB 131	Marine solar saltern	South Korea	GCF_002097535.1	NZ_CP020772.1
Bacillus sp. Y-01	Plastic contamination samples	China	GCF_003047225.1	NZ_CP024794.1
Bacillus spizizenii SW83	Seaweed	India	Unknown	CP030925.1
Bacillus subtilis subsp. subtilis BS155	Marine sediment	China	Unknown	CP029052.1
Bacillus anthracis MCCC 1A01412	Sediment	China	GCF_003410355.1	NZ_CP031643
Bacillus anthracis MCCC 1A02161	Sediment	China	GCF_003410255.1	NZ_CP031642
Bacillus amyloliquefaciens SH-B74	Marine sediment	China	GCF_003312895.1	NZ_CP030097
Lactococcus garvieae ATCC 49156	*Seriola quinqueradiata*	Japan	GCF_000269925.1	NC_015930.1
Streptococcus iniae YSFST01-82	*Paralichthys olivaceus*	South Korea	GCF_000831485.1	NZ_CP010783.1
Streptococcus iniae SF1	*Paralichthys olivaceus*	China	Unknown	CP005941.1
Lactococcus garvieae Lg2	*Seriola quinqueradiata*	Japan	GCF_000269945.1	NC_017490.1
Streptococcus parauberis KCTC 11537	*Paralichthys olivaceus*	South Korea	GCF_000213825.1	NC_015558.1
Streptococcus iniae QMA0248	*Lates calcarifer*	Australia	GCF_002220115.1	NZ_CP022392.1
Streptococcus thermophilus APC151	Marine fish (species not mentioned)	Ireland	GCF_002012365.1	NZ_CP019935.1
Lactococcus formosensis 122061	*Seriola quinqueradiata*	Japan	GCF_002355575.1	NZ_AP017373.1: NZ_AP017399.1

Streptococcus parauberis SPOF3K	*Paralichthys olivaceus*	South Korea	GCF_002900385.1	NZ_CP025420.1 NZ_CP025421.1
Jeotgalibacillus malaysiensis malaysiensis	Sea water	Malaysia	GCF_000818095.1	NZ_CP009416.1, NZ_CP009417.1
Planococcus maritimus DSM 17275	Sea water	South Korea	GCF_001687625.2	NZ_CP016538.2
Planococcus plakortidis DSM 23997	Sponge *Plakortis simplex*	India	GCF_001687605.2	NZ_CP016539.2
Planococcus donghaensis DSM 22276	Deep sea sediment	South Korea	GCF_001687665.2	NZ_CP016543.2: NZ_CP016544.1
Paenibacillus durus DSM 1735	Marine sediment	Black Sea	GCF_000756615.1	NZ_CP009288.1, NZ_CP009289.1
Paenibacillus sp. LPB0068	*Crassostrea gigas*	South Korea	GCF_001857945.1	NZ_CP017770.1: NZ_CP017771.1: NZ_CP017772.1: NZ_CP017773.1
Paenibacillus donghaensis KCTC 13049	Sea sediment	South Korea	GCF_002192415.1	NZ_CP021780.1
Paenibacillus sp. CAA11	Sediment	South Korea	GCF_003060825.1	NZ_CP028922.1
Weissella tructae WS08	*Oncorhynchus mykiss*	Brazil	GCF_000732905.1	NZ_CP007588.1
Weissella ceti WS105	*Oncorhynchus mykiss*	Brazil	GCF_000750535.1	NZ_CP009224.1
Weissella ceti WS74	*Oncorhynchus mykiss*	Brazil	GCF_000750515.1	NZ_CP009223.1
Staphylococcus aureus SJTUF_J27	Seaweed	China	GCF_001956755.1	NZ_CP019117.1
Salinicoccus sp. BAB 3246	Sea water	India	Unknown	CP020916.1
Staphylococcus delphini NCTC 12225	Dolphin (species not mentioned)	Italy	GCF_900636325.1	NZ_LR134263
Thermaerobacter marianensis DSM 12885	Mud	Pacific Ocean	GCF_000184705.1	NC_014831

Sulfobacillus acidophilus TPY	Hydrothermal vent	Pacific Ocean	Unknown	CP002901.1
Clostridium botulinum 202F	Marine sediment	USA	GCF_000789355.1	NZ_CP006903.1, NZ_CP006904.1
Thermosediminibacter oceani DSM 16646	Sea sediment	Peru	GCF_000144645.1	NC_014377.1
Carnobacterium sp. 17-4	Sea-ice	Norway	GCF_000195575.1	NC_015390.1, NC_015391.1
Desulfotomaculum reducens MI-1	Sediment	USA	GCF_000016165.1	NC_009253.1
Oscillibacter valericigenes Sjm18-20	*Corbicula japonica*	Japan	GCF_000283575.1	NC_016048.1
Kyrpidia spormannii EA-1	Sediment	Portugal	GCF_002804065.1	NZ_CP024955.1
Erysipelothrix rhusiopathiae KC-Sb-R1	*Steno bredanensis*	South Korea	GCF_003722215.1	NZ_CP033601
Enterococcus faecalis TY1	Flatfish (species not mentioned)	South Korea	GCF_003345275.1	NZ_CP031027
Petrocella atlantisensis 70B-A	Sediment	Atlantic Ocean	GCF_900538275.1	NZ_LR130778
Anoxybacter fermentans DY22613	Sediment	Pacific Ocean	GCF_003991135.1	NZ_CP016379

Table S2. Amino acid sequences of putative sactipeptide precursor predicted based on homology to known bacteriocins

Predicted bacteriocin producer	Putative amino acid sequences	Homologous	Identity (%)	E-value
Bacillus safensis KCTC 12796BP	MTRNQKEWESVSKKNLKR PGGTSIVKAAGCMGCWAS KSIAMTRVCALPHPAMRAI	Sporulation killing factor	92.73	4.79E-35
Bacillus cereus CC-1	VKNMNTPEVQPIGVTCWG CLACAACVAGYLTLVSAL SGVNTAE	Thurincin H	56.41	3.00E-14
Bacillus spizizenii SW83	MKKAVIVENKGCATCSIG AACLVDGPIPDFEIAGATG LFGLWG	Subtilosin A	100	2.40E-28
	LKLPVQQVYSVYGGKDLP KGHSHSTMPFLSKLQFLTK IYLLDIHTQPFFI	Subtilosin SboX	98	1.08E-33
Bacillus subtilis subsp. subtilis BS155	MKKAVIVENKGCATCSIG AACLVDGPIPDFEIAGATG LFGLWG	Subtilosin A	100	2.40E-28
	LKLPVQQVYSVYGGKDLP KGHSHSTMPFLSKLQFLTK IYLLDIHTQPFFI	Subtilosin SboX	98	1.08E-33
Bacillus anthracis MCCC 1A01412	MFKIFCVFLENMGSYTLVA PHNTEKEVGQMETPVVQP RDWACWSCLVCTACSVEL LNLVTAANGASTAS	Thurincin H	92.5	9.99E-24
Staphylococcus delphini NCTC 12225	MEQGVMVSNKGCSACAV GAVCLADGPIPDFEVAGIT GTFGIAS	Subtilosin A	63.415	1.72E-18

Table S3. Amino acid sequences of putative lanthipeptide precursors predicted based on homology to known bacteriocins

**Predicted bacteriocin producer**	**Putative amino acid sequences**	**Homologous**	**Identity (%)**	**E-value**
Geobacillus kaustophilus HTA426	LSKNRTTLYNLILQVNLF CTPGCITGTLMCLTQNSC VSCNSCIRC	Geobacillin I	92,857	9,29E-16
	MAKLDDFDLDIVVKKQD NIVQPNITSKSLCTPGCIT GILMCLTQNSCVSCNSCI RC	Salivaricin D	50	2.00E-13
Bacillus weihaiensis Alg07	MIEMSKEVKVTSEEIIEAL KNQDVRAKFEGITHPSGK ALNELSEEELAAIQGASD VQPETTPLCVGVIIGLTTS IKIC	Cerecidin	76,316	1,87E-37
Bacillus velezensis 9912D	MKKEFLALELMTEEELK ELAGGSEATPMTVTPTTI TIPISLAGCPTTKCASIVSP CND	Lichenicidin A2	40,816	4,10E-08
Paenibacillus durus DSM 1735	LKMLTGGAGDVNPETTIL TTSSWTCITAGVTVSASL CPTTKCTSQC	Lichenicidin A2	84,783	3,18E-22
	MMISMKNSKIRNEFLKTS KTVGVVTEEELKMLTGG AGDVNPETTILTTSSWTCI TAGVTVSASLCPTTKCTS QC	Lichenicidin A2	69.863	1.60E-31
	MSGIPSLLIFSIKGKGAIT MYDKALVTAWKNPMAR KGLGAIHHPSGDVLAELK EEDLQDFTGGFDTNTLTT STSILISMTLGNNGWVCT ATKECMPSCN	Lichenicidin A1	48.75	4.34E-25

Paenibacillus sp. CAA11	LKGVRQMAKNSMFDLDVQVTSVKEEASAQLAQSRLICTPGSCDGDRCWLTTFKGCPTYGPKCDI	Streptin	36,364	6,65E-07
Enterococcus faecalis TY1	MENLSVVPSFEELSVEEMEAIQGSGDVQAETTPVCAVAATAAASSAACGWVGGGIFTGVTVVVSLKHC	Cytolysin ClyLl	100	6,88E-46
	VLNKENQENYYSNKLELVGPSFEELSLEEMEAIQGSGDVQAETTPACFTIGLGVGALFSAKFC	Cytolysin ClyLs	98.413	2.28E-43
Bacillus spizizenii SW83	MEKNNIFDLDINKKMESTSEVSAQTWATIGKTIVQSVKKCRTFTCGCSLGSCSNCN	Subtilomycin	100	1,92E-38

Table S4. Amino acid sequences of putative LAP precursors predicted based on homology to known bacteriocins

Predicted bacteriocin producer	Putative amino acid sequences	Homologous	Identity (%)	E-value
Streptococcus iniae YSFST01-82	MLQFTSNILATSVAETTQVAPGGCCCCCCTCCVAVNVGSGSAQGGSGTPAPAPK	Streptolysin	85,714	5,29E-12
Streptococcus iniae SF1	MLQFTSNILATSVAETTQVAPGGCCCCCCTCCVAVNVGSGSAQGGSGTPAPAPK	Streptolysin	85,714	5,29E-12
Streptococcus iniae QMA0248	MLQFTSNILATSVAETTQVAPGGCCCCCCTCCVAVNVGSGSAQGGSGTPAPAPK	Streptolysin	85,714	5,29E-12

Table S5. Amino acid sequences of putative head-to-tail cyclized peptide precursors predicted based on homology to known bacteriocins

Predicted bacteriocin producer	Putative amino acid sequences	Homologous	Identity (%)	E-value
Geobacillus kaustophilus HTA426	MSLLALVAGTLGVSQSIATTVVSIVLTGSTLISIILGITAILSGGVDAILEIGWSAFVATVKKIVAERGKAAAIAW	Circularin A	45,07	1,23E-14
Bacillus sp. Pc3	MMNLVKSNKKSFILFGAALAAATLVYALLLTGTELNVAAAHAFSANAELASTLGISTAAAKKAIDIIDAASTIASIISLIGIVTGAGAISYAIVATAKTMIKKYGKKYAAAW	Amylocyclicin	100	1,19E-73
Bacillus velezensis 9912D	MMNLVKSNKKSFILFGAALAAATLVYALLLTGTELNVAAAHAFSANAELASTLGISTAAAKKAIDIIDAASTIASIISLIGIVTGAGAISYAIVATAKTMIKKYGKKYAAAW	Amylocyclicin	100	1,19E-73
Bacillus amyloliquefaciens SH-B74	MMNLVKSNKKSFILFGAALAAATLVYALLLTGTELNVAAAHAFSANAELASTLGISTAAAKKAIDIIDAASTIASIISLIGIVTGAGAISYAIVATAKTMIKKYGKKYAAAW	Amylocyclicin	100	1,19E-73

Kyrpidia spormannii EA-1	VGGLVFDLMGFLGVGNW VAQQIVSLINQFGWAIITM SIITTILSGGSLSVWTASAD YIVAVVLNYLKRNLWLQ AIAW	Uberolysin	37,838	4,98E-12

Table S6. Amino acid sequences of putative lasso peptide precursors predicted based on homology to known bacteriocins

Predicted bacteriocin producer	Putative amino acid sequences	Homologous	Identity (%)	E-value
Bacillus cereus CC-1	MKKDWTIPTLEVLDINMT MAGPGLKTPDAVQPDIDE VVHYS	Paeninodin	76,19	4,24E-08
Paenibacillus crassostreae LPB0068	VKTMKKEWNIPALEILNVS MTMAGPGNARPDGVQPDP DETIHHTS	Paeninodin	60,87	1,12E-07

Table S7. Amino acid sequences of proteusin peptide precursors predicted based on homology to known bacteriocins

Predicted bacteriocin producer	Putative amino acid sequences	Homologous	Identity (%)	E-value
Paenibacillus crassostreae LPB0068	MTQMALFQTQVINKAWED PSFKALLLTDPKKAIKDAL GVIIPEHIKLRTLEESSDEFY LVLPSSPSNNTPDNVQSKA MW	PoyA	30,303	1,36E-10
Paenibacillus sp. CAA11	VILMSTQGALLQSQVVQK AWQDPSFKTKLLSDPKAA LQEFLGVELPDHIKVKAVE EQSDEFFIVLPPNPSKALAP DVKPLVMWN	PoyA	32,812	4,05E-11

Table S8. Amino acid sequences of proteusin ComX precursors predicted based on homology to known bacteriocins

Predicted bacteriocin producer	Putative amino acid sequences	Homologous	Identity (%)	E-value
Bacillus velezensis 9912D	MEEITVKQDIVHYLMENPLV LTKLACGEASLIGVPDKLLPA IVEIFERDFSQSKKCRGIFWEQ	ComX1	96,825	4,69E-44
Bacillus subtilis subsp. subtilis BS155	MQEIVGYLVKNPEVLDEVME GRASLLNIDKDQLKSIVDAFR GLQIYTNGNWVPS	ComX4	46,154	2,18E-08

Table S9. Amino acid sequences of lysine-to-tryptophan crosslink and autoinducing peptides precursors predicted based on homology to known bacteriocins

Predicted bacteriocin producer	Putative amino acid sequences	Homologous	Identity (%)	E-value
Staphylococcus aureus SJTUF_J27	MKKLLNKVIELLVDFFNSIGY RAAYINCDFLLDEAEVPKELT QLHE	Autoinducing Peptide III	100	3,49E-31
	MSCLILRIFILIKEGVISMAQDI ISTIGDLVKWIIDTVNKFTK	delta-lysin I	80	1,23E-09
Streptococcus thermophilus APC151	VLKRNIITVSYSKGVPNMS KELEKVLESSSMAKGDGW KVMAKGDGWE	Streptide	100	2,03E-17

Table S10. Amino acid sequences of class II bacteriocin precursors predicted based on homology to known bacteriocins

Predicted bacteriocin producer	Putative amino acid sequences	Homologous	Identity (%)	E-value
Bacillus sp. Pc3	VLEMNFKKVLTGSALSL ALLMSAAPAFAASPTASA SAENSPISTKADAGINAIK LVQSPNGNFAASFVLDGT KWIFKSKYYDSSKGYWV GIYESVDK	LCI	93,478	1,50E-29
	LNGGELDVLKYFLTQGPF AVLFTWLLIYVMKSNRE RESRLQDLLDKFSDKYDV IIDKIDRLEEKFRGRE	UviB	33,333	8,16E-12
Bacillus safensis KCTC 12796BP	VIPMEVDVVQNLMTQGP FAVLFCWILFYVLNTTKE RENKLNEQIEAQNDVLA KFSEKYDVVIDKLDKIER NLK	UviB	37,5	2,28E-12

Bacillus velezensis 9912D	VFEMKFKKVLTGSALSLA LLMSAAPAFAASPTASSS VENSPISTKADVGINAIKL VQSPDGNFAASFVLNGTT WIFKSKTYDSGRGIWIGV YESVDK	LCI	73,913	1,10E-23
Bacillus amyloliquefaciens SH-B74	MKFKNVLTGSALSLALL MSAAPAFAASPTASASAE NSPISTKADAGINAIKPVP SPNGIFAASFELNGTTWIF KYKYYDSSKGYWVGIYE SVDK	LCI	78,261	1,73E-22
Streptococcus iniae YSFST01-82	MKKISKIWAVGLVAASLS FGSIAYAESISVAGGTWN YGYGVGQAYSHYKHDY NNHGAKVVNSNNGVKD YKNAGPGVWAKASIGTV WDPATFYYNPTGFYSN	Lactococcin 972	42,105	3,44E-18
	LHEVDLASVDGGKINWG SVAGNCVGGAIITGAFSG LLYILDAGIGCVVGSGQSI INEL	Thermophilin 13	62,069	3,13E-15
Streptococcus iniae SF1	MKKISKIWAVGLVAASLS FGSIAYAESISVAGGTWN YGYGVGQAYSHYKHDY NNHGAKVVNSNNGVKD YKNAGPGVWAKASIGTV WDPATFYYNPTGFYSN	Lactococcin 972	42,105	3,44E-18
	LHEVDLASVDGGKINWG SVAGNCVGGAIITGAFSG LLYILDAGIGCVVGSGQSI INEL	Thermophilin 13	62,069	3,13E-15
Streptococcus iniae QMA0248	MKKISKIWAVGLVAASLS FGSIAYAESISVAGGTWN YGYGVGQAYSHYKHDY NNHGAKVVNSNNGVKD YKNAGPGVWAKASIGTV WDPATFYYNPTGFYSN	Lactococcin 972	42,105	3,44E-18
	LHEVDLASVDGGKINWG SVAGNCVGGAIITGAFSG LLYILDAGIGCVVGSGQSI INEL	Thermophilin 13	62,069	3,13E-15
Streptococcus thermophilus APC151	MATQTIENFNTLDLETLA SVEGGVCSWGGFAKQGV ATGVGNGLRLGIKTRTW	BlpU	98,684	6,38E-50

	QGAVAGAAGGAIVGGVG YGATCWW			
	MATQTIENFNTLDLETLA SVEGGLSCDEGMLAVGG LGAVGGPWGAVGGVLV GAALYCF	BlpD	100	1.88E-38
	MATQTIENLNTLDLETLA SVEGGGCSWGGAGGATV QGAIGGAIGGAFGGNVVL PVVGSVPGYLAGGVLGG AGGTVAYGATCWWS	BlpK	92.857	9.09E-49
Lactococcus formosensis 122061	MENNNYTVLSDEELQEID GGIGGALGNALNGLGTW ANMMNGGGFVNQWQVY ANKGKINQYRPY	Garvieacin Q	90	2,75E-09
Jeotgalibacillus malaysiensis malaysiensis	MDNSFIEYLATQGIFAVFF GLLFLYVLRTSKKREEEY KKMIALQQENLPEIKKLL TILSKEK	UviB	43,396	1,51E-12
Paenibacillus durus DSM 1735	MTEADVLKYFLTQGPFA VLFVWLLIYVMKTNSKR EGRLQDLLDKFSDKYDVI IGELREMKERFPRGKE	UviB	34,921	2,69E-13
Staphylococcus delphini NCTC 12225	MKNKILSTLVAGLLLIGIG SSSIVSAATIYTDGGTWN YGVGSKYVWSYYSNNYR YHSSTAIGKTRSFSGFRKP GVRALASSEKRWWWRN EAYYNVY	Lactococcin 972	44,898	1,43E-26

Table S11. Amino acid sequences of class III bacteriocin precursors predicted based on homology to known bacteriocins

Predicted bacteriocin producer	Putative amino acid sequences	Homologous	Identity (%)	E-value
Bacillus spizizenii SW83	MVMKVFEAKTLLSEATDRAKEY KELRTQMVNLRKALKSVADLSD SEFSGKGASNIKAFYHDHVGVTD QWIDYIDMKIAFFNSIAGAAEDK GLSDAYIEESFLEHELANAHKKS KSIMSEQKKAMKDILNDIDDILPL DLFSTETFKNELADANDKRKKTL EKLDTLDEDLKTEYTLSEPNEQFI KSDFQKLQEATGKGKNATPIHY NAKAYRESDIHKKKGDIEKRTEA YLKIKKEEAKEREIEKLKKKLAD GVSDPDEYLEIAKKIGYENLEPT QLQYVVQLEQAKQLEEVGETTW EVLKGVGVGLYDVAKDTVTGV KDLAVGAWEFSQLSEEQKLAKTI STLLKTPSYAKIIWTNIADSWND KMVNGDAYSRSHYITYVVGSLV GLKGAGSVVKVTSKLGKAGAAK VDKVLEAGEKATTKHVKTGIEK GKNFINSFPKNEYEPALVGIAQDI GNTHNVKNTPLLKKIIEESKESSI LFTKRNIPGVATGKVTPSRGLQL QERLMYSNGKLGVIPQEVRKQL LGKEFKSFDEFRKEFWKSVANTE YANEFNKNNIARMKNGNSPIAPK KEHYGKHKSYILHHKQPIDKGG DVYNLDNLIITSPKMHQEILDPA YHFGKKGSKN	Colicin	40,602	5,03E-27

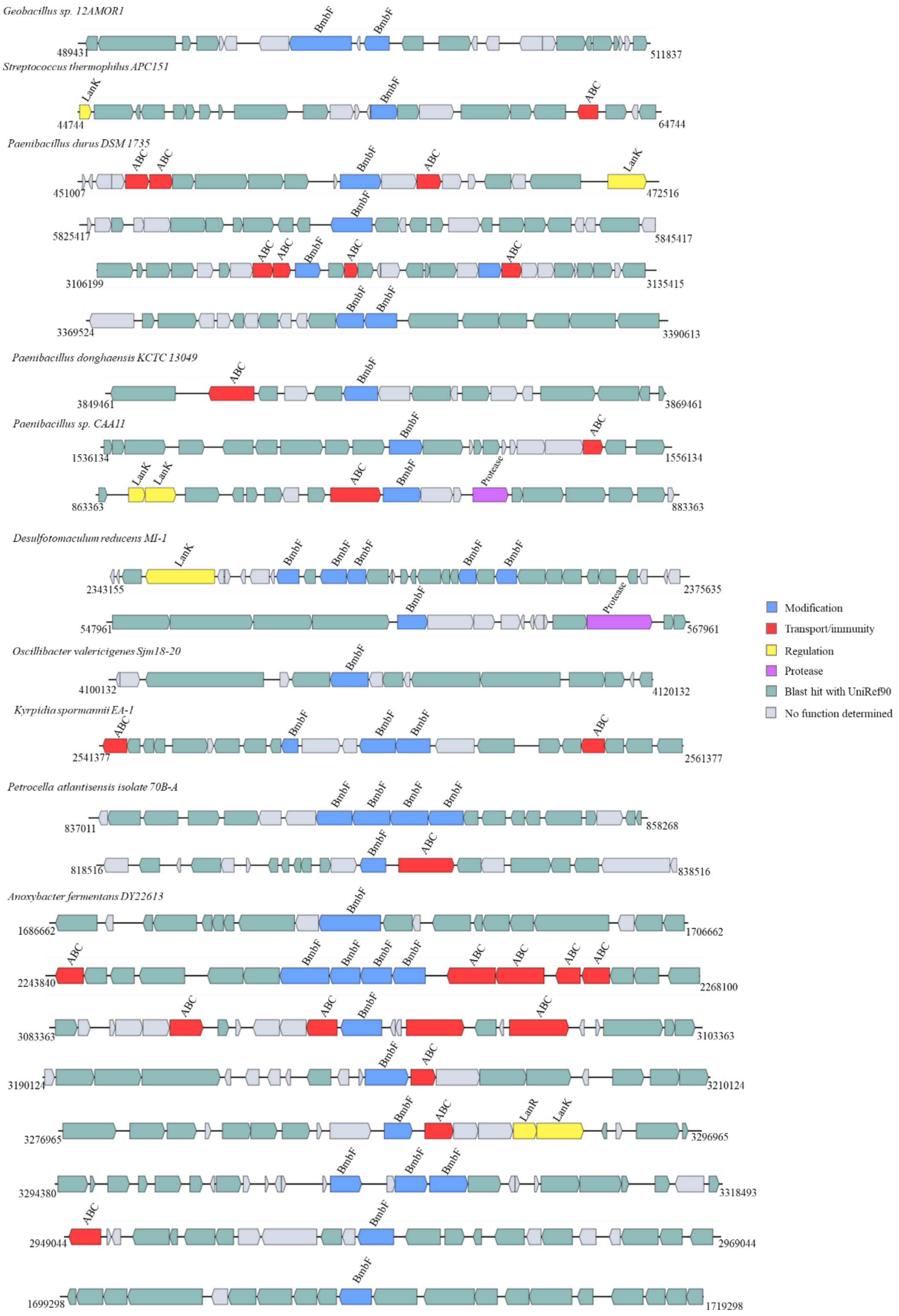

Figure S1. Gene cluster organization of sactipeptides identified in the selected *Bacillota* genomes based on the presence of associated context genes (examined by BAGEL4)

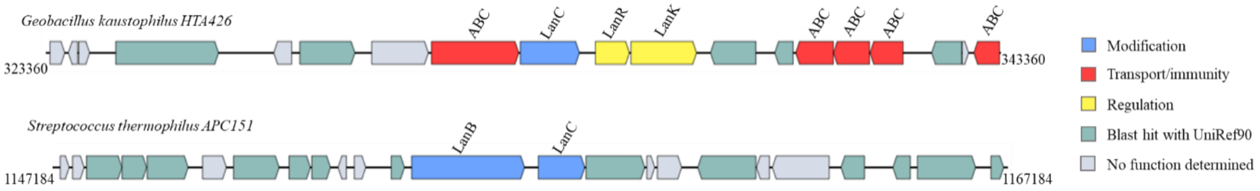

Figure S2. Gene cluster organization of lanthipeptides identified in the selected *Bacillota* genomes based on the presence of associated context genes (examined by BAGEL4)

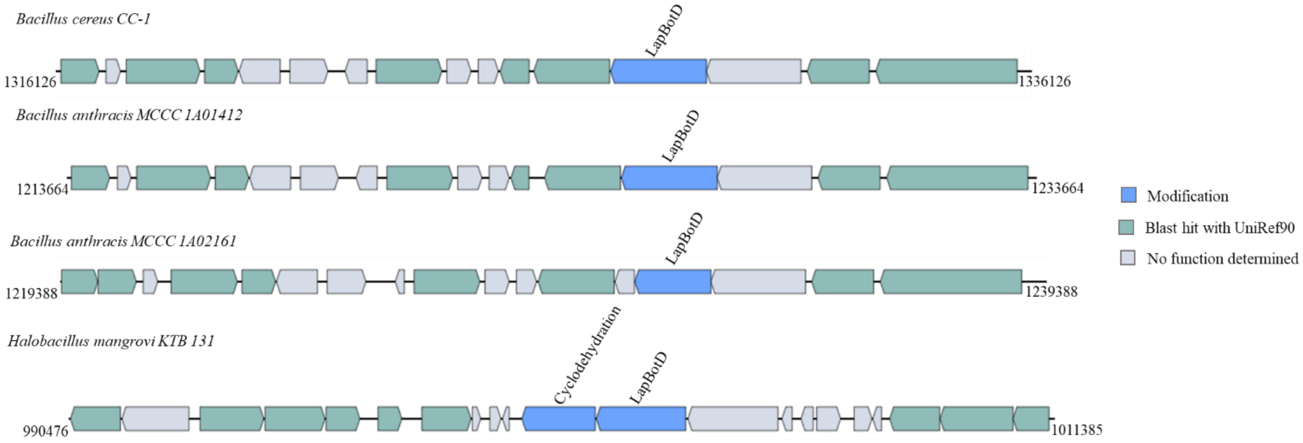

Figure S3. Gene cluster organization of linear azole-containing peptides identified in the selected *Bacillota* genomes based on the presence of associated context genes (examined by BAGEL4)

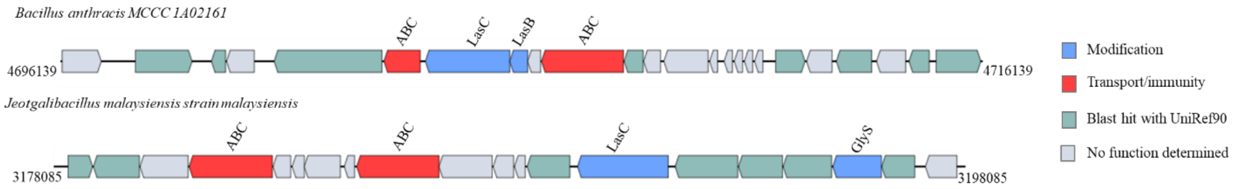

Figure S4. Gene cluster organization of lasso peptides identified in the selected *Bacillota* genomes based on the presence of associated context genes (examined by BAGEL4)
